# Bioclojure: a functional library for the manipulation of biological sequences

**DOI:** 10.1093/bioinformatics/btu311

**Published:** 2014-05-02

**Authors:** Jordan Plieskatt, Gabriel Rinaldi, Paul J. Brindley, Xinying Jia, Jeremy Potriquet, Jeffrey Bethony, Jason Mulvenna

**Affiliations:** ^1^Department of Microbiology, Immunology and Tropical Medicine, ^2^Research Center for Neglected Diseases of Poverty, School of Medicine and Health Sciences, George Washington University, Washington, DC, 20052, USA, ^3^QIMR Berghofer Medical Research Institute, Infectious Disease and Cancer and ^4^The University of Queensland, School of Biomedical Sciences, Brisbane, Queensland, 4072, Australia

## Abstract

**Motivation:** BioClojure is an open-source library for the manipulation of biological sequence data written in the language Clojure. BioClojure aims to provide a functional framework for the processing of biological sequence data that provides simple mechanisms for concurrency and lazy evaluation of large datasets.

**Results:** BioClojure provides parsers and accessors for a range of biological sequence formats, including UniProtXML, Genbank XML, FASTA and FASTQ. In addition, it provides wrappers for key analysis programs, including BLAST, SignalP, TMHMM and InterProScan, and parsers for analyzing their output. All interfaces leverage Clojure’s functional style and emphasize laziness and composability, so that BioClojure, and user-defined, functions can be chained into simple pipelines that are thread-safe and seamlessly integrate lazy evaluation.

**Availability and implementation:** BioClojure is distributed under the Lesser GPL, and the source code is freely available from GitHub (https://github.com/s312569/clj-biosequence).

**Contact:** jason.mulvenna@qimrberghofer.edu.au or jason.mulvenna@qimr.edu.au

## 1 INTRODUCTION

Functional programming is a programming style that treats computation as the evaluation of mathematical functions (Hudak, 1989). In its purest form, functional programming removes the need for variable assignment by using immutable data structures that eliminate the use of state and side effects (Backus, 1978). This ensures that functions will always return the same value given the same input. This greatly simplifies debugging and testing, as individual functions can be assessed in isolation regardless of a global state. Immutability also greatly simplifies concurrency and facilitates leveraging of multi-core computing facilities with little or no modifications to functionally written code. Accordingly, as a programming style, functional programming offers advantages for software development, including (i) brevity, (ii) simple handling of concurrency and (iii) seamless integration of lazy evaluation, simplifying the handling of large datasets. Clojure is a Lisp variant that encourages a functional style of programming by providing immutable data structures, functions as first-class objects and uses recursive iteration as opposed to state-based looping (Hickey, 2008). Clojure is built on the Java virtual machine (JVM), and thus, applications developed using BioClojure can be compiled into Java byte code and ran on any platform that runs the JVM. Moreover, libraries constructed using Clojure can be called in Java programs and, conversely, Java classes and methods can be called from Clojure programs, making available a large number of third-party Java libraries. BioClojure aims to leverage the tools provided by Clojure to provide a functional interface with biological sequence data and associated programs. BioClojure is similar in intent to other bioinformatics packages such as BioPerl (Stajich *et al.*, 2002), BioPython (Cock *et al.*, 2009), Bio++ (Dutheil *et al.*, 2006) and BioJava (Prlić *et al.*, 2012) but differs from these bioinformatics software libraries in its embrace of the functional style. With the decreasing cost of biological analyses, for example, next-generation sequencing, biologists are dealing with greater amounts of data, and BioClojure is an attempt to provide tools, emphasizing concurrency and lazy evaluation, for manipulating these data.

## 2 METHODS

BioClojure source code and extensive documentation are available via GitHub (https://github.com/s312569/clj-biosequence). The library is available as a Java jar file from Clojars (https://clojars.org/clj-biosequence) and can be easily incorporated into Clojure projects using lein (http://leiningen.org/). BioClojure is organized into name-spaces (modules), each either providing access to a particular sequence format, providing a wrapper to key programs, BLAST and SignalP, or providing other functionality, for example, indexing and biological alphabets. When designing functions contained within BioClojure, efforts have been made to maintain laziness and composability. This, in combination with the Clojure threading macros, facilitates the construction of analysis pipelines that can process sizeable files using minimal memory.

### 2.1 The core module

The core module provides core functions such as DNA and protein alphabets as well as translation, key accessors and file functions. More importantly, it establishes a framework for parsing sequence files using the functions ‘bs-reader’ and ‘biosequence-seq’. Almost every module in BioClojure implements these functions to access its particular sequence format or type of data. When used in combination with the in-built Clojure macro ‘with-open’, these functions provide lazy access to on-disk data. For example, a simple pipeline to translate a file of nucleotide sequences in six-reading frames would use these functions in the following way:

user> (with-open [r (bs-reader fasta-file)]
     (->> (biosequence-seq r)
        (mapcat #(six-frame-translation %))
        realized?))
false

This code provides a lazy sequence of FASTA protein sequences representing the six-frame translation of nucleotide sequences from ‘FASTA-file’. The final call to ‘realized?’ merely illustrates the lazy nature of the calculation. The resulting sequences can be sent to the file using the BioClojure function ‘biosequence->file’ or further processed using BioClojure and/or user-defined functions. The use of immutable objects and stateless iteration can lead to simple and easily understandable code. A simple example of this is the following code, which returns counts for biological process GO terms from secreted proteins in the UniProt Human proteome dataset:

(with-open [r (bs-reader up-hs-proteome)]
  (->> (biosequence-seq r)
    (filter #(some (fn [x] (= “Secreted”
                   (:text x)))
          (subcellular-location %)))
    (mapcat bp-go-terms)
    frequencies))
{“neurotrophin TRK receptor signaling pathway”
 36, … .

The defined interfaces of BioClojure are designed to be lazy and composable in this way, and thus, more complex examples of these simple lazily evaluated pipelines can be developed.

### 2.2 Sequence formats

At present, BioClojure supports sequence data formatted as Uniprot XML, Genbank XML FASTA and FASTQ. For each format, apart from parsers, BioClojure provides accessors specific to that format (see https://github.com/s312569/clj-biosequence for detailed documentation). BioClojure also provides functions for remote searching and sequence retrieval from UniProt and GenBank. For mapping of identification numbers, BioClojure provides the ‘id-convert’ function that uses the UniProt accession mapping service to convert accession numbers from one database format to another. Integration of diverse file formats with the structure provided by the core module is implemented using Clojure protocols; therefore, implementation of modules for new formats is facile, with additional formats, in particular GFF and GTF, expected to be supported in the near future.

### 2.3 Application wrappers

In addition to sequence data, BioClojure also provides wrappers for running BLAST, SignalP, THMHH and Interproscan as well as parsers for their output. Once again, integration of these tools with BioClojure emphasizes lazy evaluation and composability, which simplifies integration of the tools with other parts of BioClojure.

### 2.4 Persistence

The ‘index’ module provides functions for producing compressed and indexed files. An indexed file implements ‘biosequence-seq’ and thus can be used the same way as described above, but without the requirement for using ‘with-open’ or ‘bs-reader’. Indexed files also provide rapid random access to indexed sequences using the ‘get-biosequence’ function.

### 2.5 Concurrency

One of the primary motivations for using Clojure is the built-in support for concurrent operations. One simple example of this support is the ‘pmap’ function. The Clojure function ‘map’ serially applies a function to a list of inputs, returning a list of the outputs, and ‘pmap’ performs the same operations using multiple threads. If the computational cost of the applied function outweighs the coordination costs, significant performance gains are possible, as shown below using the SwissProt database:

user> (time (with-open [r (bs-reader swissprot)]
     (last (map protein-charge
           (biosequence-seq r)))))
“Elapsed time: 101232.610534 msecs”
5.778330187793381
user> (time (with-open [r (bs-reader swissprot)]
     (last (pmap protein-charge
           (biosequence-seq r)))))
“Elapsed time: 30552.548286 msecs”
5.778330187793381

In practice, ‘pmap’ initiates a limited number of threads, based on the number of cores; therefore, for large datasets, or asynchronous calls, a finer-grained control over the number of threads and their behavior can be obtained using Clojure’s software transactional memory, agent and atom systems.

## 3 CONCLUSION

BioClojure is a functional software library specifically designed for parsing and processing biological sequence data. It provides a lazy and thread-safe framework for accessing and streaming these data while using minimal amounts of memory. Presently, we extensively use the library for the annotation of nucleotide and peptide sequences arising from next-generation sequencing and the proteomic analysis of complex protein mixtures. We plan to extend the functionality of the library by incorporating modules for phylogenetic and proteomic analyses, and we welcome contributions from the community.

*Funding*: National Health and Medical Research Council, Australia (grant number 1051627), as well as award R01CA155297 from the National Cancer Institute.

*Conflict of Interest*: none declared.
